# Assessing Response Using ^99**m**^Tc-MIBI Early after Interstitial Chemotherapy with Carmustine-Loaded Polymers in Glioblastoma Multiforme: Preliminary Results

**DOI:** 10.1155/2014/684383

**Published:** 2014-03-27

**Authors:** D. Cecchin, I. Schiorlin, A. Della Puppa, G. Lombardi, P. Zucchetta, V. Bodanza, M. P. Gardiman, G. Rolma, A. C. Frigo, F. Bui

**Affiliations:** ^1^Nuclear Medicine Service, Department of Medicine (DIMED), University Hospital, 35128 Padova, Italy; ^2^Department of Neurosurgery, University Hospital, 35128 Padova, Italy; ^3^Department of Medical Oncology 1, IOV, IRCCS, Venetian Oncology Institute, 35128 Padova, Italy; ^4^Surgical Pathology & Cytopathology Unit, Department of Medicine (DIMED), University Hospital, 35128 Padova, Italy; ^5^Neuroradiology Unit, University Hospital, 35128 Padova, Italy; ^6^Biostatistics, Epidemiology and Public Health Unit, Department of Cardiac, Thoracic and Vascular Sciences, University Hospital, 35128 Padova, Italy

## Abstract

*Introduction*. Early signs of response after applying wafers of carmustine-loaded polymers (gliadel) are difficult to assess with imaging because of time-related imaging changes. ^99m^Tc-sestamibi (MIBI) brain single-photon emission tomography (SPET) has reportedly been used to reveal areas of cellularity distinguishing recurrent neoplasm from radionecrosis. Our aim was to explore the role of MIBI SPET in assessing response soon after gliadel application in glioblastoma multiforme (GBM). *Methods*. We retrospectively reviewed the charts on 28 consecutive patients with a radiological diagnosis of GBM who underwent MIBI SPET/CT before surgery (with intracavitary gliadel placement in 17 patients), soon after surgery, and at 4 months. The area of uptake was selected using a volume of interest that was then mirrored contralaterally to obtain a semiquantitative ratio. *Results*. After adjusting for ratio at the baseline, the effect of treatment (gliadel versus non-gliadel) was not statistically significant. Soon after surgery, however, 100% of patients treated with gliadel had a decreased ratio, as opposed to 62.5% of patients in the non-gliadel group (*P* = 0.0316). The difference between ratios of patients with radical versus partial resection reached statistical significance by a small margin (*P* = 0.0528). *Conclusions*. These data seem to suggest that the MIBI ratio could be a valuable tool for monitoring the effect of gliadel early after surgery.

## 1. Introduction

Interstitial chemotherapy with carmustine-loaded polymers (aka “gliadel wafers,” MGI Pharma, Bloomington, MN, USA) has been recognized [1] as a valid therapeutic option in newly diagnosed [2–5] gliomas and a debatable option in recurrent [6, 7] high-grade gliomas (HGG).

Early signs of response after applying gliadel wafers are very difficult to assess using magnetic resonance imaging (MRI) because of the imaging changes occurring with time at wafer level and in the adjacent brain tissue [8], which can easily be mistaken for an abscess. MRI of early changes (especially a month after wafer implantation) is unable to clearly distinguish between postoperative changes and residual neoplasm and provides no information for predicting survival or progression-free interval. Pseudoprogression has also been reported, using MRI, within the first two months after gliadel placement [9, 10].

On the other hand, ^99m^Tc-sestamibi (MIBI) brain single-photon emission tomography/computed tomography (SPET/CT) can reveal areas of cellularity in high-grade gliomas [11], thus distinguishing recurrent neoplasm from radionecrosis after radiotherapy [12, 13]. MIBI (a substrate for P-glycoprotein and the multidrug resistance protein) has also been claimed to predict survival and early response or resistance to chemotherapy [14, 15].

To our knowledge, there are currently no data on the use of MIBI SPET/CT after applying gliadel at any time (and early after surgery in particular). The object of this study was thus a retrospective review of the charts on 17 patients with histologically proven glioblastoma multiforme (GBM) who underwent SPET/CT using MIBI (before surgery, soon after surgery, and 4 months after gliadel application), the well-known Stupp protocol (radiotherapy plus concomitant and adjuvant temozolomide) [16], and gliadel implantation. We also reviewed the charts on 11 comparable patients not implanted with gliadel and compared their outcomes with those of the gliadel group.

## 2. Methods

### 2.1. Patients

We retrospectively reviewed a total of 28 consecutive patients (20 males and 8 females, median age 61.5 years, range 39–84 years) with a radiological (MRI) diagnosis of GBM, who had SPET/CT using MIBI at the nuclear medicine unit of the University of Padova before undergoing surgery. All patients were treated surgically for GBM using an MRI neuro-navigational method, with neurophysiological monitoring where necessary. The diagnosis of GBM was confirmed at histology in all cases.

Intracavitary gliadel wafers were inserted in 17/28 patients (13 males and 4 females; median age 58 years, range 39–69 years) who formed the “gliadel group,” while the other 11/28 patients (7 males and 4 females; median age 67 years, range 51–84 years) served as controls (“non-gliadel group”). The controls were cases of multifocal tumour, or disease extending across the corpus callosum, or requiring a large opening in the ventricle, or patients who refused gliadel wafer placement.

In the gliadel group, 11/17 patients underwent gross-total resection, while resection was partial in 6/17 because intraoperative monitoring showed that eloquent cortical areas were involved. In the non-gliadel group, 7/11 had gross-total resections and 4/11 had partial resections. Six of the 17 patients in the gliadel group were classified as first diagnoses of HGG and 11/17 as recurrent cancers; all the non-gliadel controls were first diagnoses.

### 2.2. Imaging

SPET/CT using MIBI started 15 min after intravenous injection of 740 MBq of MIBI (Bristol-Myers Squibb Medical Imaging, Brussels, Belgium). MIBI preparation and quality control were according to the manufacturer's instructions. A dual-head *γ*-camera (Infinia Hawkeye; GE Healthcare, Chalfont St. Giles, UK) equipped with a crystal 3/8ths of an inch thick and a low-energy, high-resolution collimator was used to acquire SPET/CT. Emission data were acquired first (“head first,” supine position, step-and-shoot mode, 45 s/step—128 × 128 matrix size—energy window of 140 ± 10 KeV and scatter correction window 120 ± 5% keV). The transmissive CT (2.5 mA, 140 kV) field of view was selected on emission images, which were reconstructed (using an algorithm for resolution recovery and scatter correction) at a Xeleris workstation using 4 iterations for ordered subset expectation maximisation (OSEM-10 subsets) and 20 iterations for maximum likelihood expectation maximisation (MLEM). A 3D Butterworth post-filter was applied (cut-off frequency 0.65 cycle/cm, order 10) to each study. Attenuation was corrected using transmissive images. An average effective dose of 0.1 mSv was estimated for CT head scanning using Infinia Hawkeye [17]. An effective dose of 9.0*E* − 03 mSv/MBq was estimated for ^99m^Tc-MIBI in resting individuals [18].

SPET/CT using MIBI was performed

- before surgery in all cases;
- early after surgery in 27/28 patients (17/17 gliadel group; 10/11 non-gliadel group), after a mean 1.16 months (min 0.67, max 2.17 months); 1 non-gliadel control patient refused the procedure at this time point and was not considered for the purposes of the statistical analysis;
- at 4 months in 19/28 patients (10/17 gliadel group; 9/11 non-gliadel group), after a mean of 3.77 months (min 2.43, max 5.13 months); 9/28 cases were not acquired at this time point.

Four patients (2 in each group) were not considered in the statistical analysis because

- MIBI was impossible to interpret in one patient (gliadel-group) due to the patient moving excessively (even when the acquisition was repeated);
- the interval between presurgical MIBI and surgery was too long in one patient (gliadel group), that is, 138 days versus a mean of 8.96 days (min 1 day; max 61 days);
- one neoplasm (non-gliadel group) was not visible on presurgical MIBI, probably due to resolution limits, so any acquisition early after surgery became pointless;
- one periventricular neoplasm (non-gliadel group) revealed only a faint uptake and it was impossible to draw VOIs without including physiological activity in the choroid plexus.

A summary of the statistically analysed data is given in Table 1.

### 2.3. Image Analysis

Transaxial, sagittal, and coronal views (attenuation-corrected (AC) and nonattenuation-corrected (NAC) images) were obtained for each study and analysed qualitatively and semiquantitatively.

For each study and time period, the area of MIBI uptake was selected (on AC images) using a volume of interest (VOI) that was then corrected manually on each transaxial slice to ensure that only specific uptake was included (excluding any activity in the skull or choroid plexus, for instance). The resulting “tumoural VOI” was then mirrored contralaterally on healthy brain to obtain the following ratio (an estimate of the lesion's “cellularity”), which is a dimensionless quantity:

(1) $$\begin{array}{c} \text{Ratio}=\frac{\left(\text{counts}/\text{pixel}\;\text{in}\;\text{tumoural}\;\text{VOI}\right)}{\left(\text{counts}/\text{pixel}\;\text{in}\;\text{VOI}\;\text{in}\;\text{healthy}\;\text{brain}\right)}. \end{array}$$

### 2.4. Surgical Technique and Sample Collection

Bis-chloroethylnitrosourea (BCNU) wafers were implanted after surgical removal of the neoplasm and intraoperative pathological confirmation of HGG. An accurate haemostasis was achieved before implanting the wafers in all cases. The surgical cavity was checked to see if there was any communication between the surgical cavity and the cerebral ventricles: if so, the wafers were only inserted when this defect was smaller than 10 mm [19]. The number of wafers inserted depended on the size of the surgical cavity, but it was never more than 8 (mean 6; min. 4, max. 8).

### 2.5. Pathological Analysis

Frozen sections were examined intraoperatively to confirm the provisional diagnosis of HGG in all cases before inserting any wafers to rule out patients with metastases or other neoplasms. The final histological diagnosis was subsequently established on formalin-fixed and paraffin-embedded surgical specimens. Fresh surgical specimens were cut into fragments, immersed in 10% buffered formalin, and left for 24 hours. After fixation, specimens were dehydrated in graded alcohol, cleared in xylene, and embedded in paraffin; histology was performed on 3 mm thick sections stained with haematoxylin & eosin.

Additional immunohistochemical stains, such as GFAP, neurofilament, synaptophysin, and the biomarkers MIB1 and p53, were used to reach the correct diagnosis.

### 2.6. Chemotherapy

Gliadel wafers (MGI Pharma, Bloomington, MN, USA), an FDA-approved medication for newly diagnosed malignant glioma and for recurrent GBM, were used as an adjunct to surgery in 17/28 patients.

Gliadel is a hydrophobic copolymer matrix (wafer) impregnated with BCNU that is inserted in the surgical cavity after tumour resection. The wafers are designed to release the chemotherapeutic agent over a period of 2-3 weeks at a near constant rate (by hydrolysis of the polymers caused by the aqueous environment of the brain).

Four to 6 weeks after the surgical procedure, all patients with a first diagnosis of HGG were administered the “Stupp protocol” with a combination of radiation therapy and temozolomide, followed, after a 4-week rest period, by temozolomide alone. Patients with recurrent GBM received systemic chemotherapy with fotemustine 100 mg/m^2^ weekly for 3 consecutive weeks followed by a 5-week rest period.

### 2.7. Statistical Analysis

The ratio (counts/pixel in tumoural VOI)/(counts/pixel in VOI in healthy brain) early after surgery was analysed using a one-way analysis of covariance (ANCOVA) model considering:

- the baseline ratio as covariate and the treatment factor (gliadel/no gliadel);
- the baseline ratio as covariate and the completeness of resection factor (yes/no);
- the baseline ratio as covariate and the first diagnosis factor (first diagnosis/relapsing disease) in the gliadel-treated patients alone.

Checks were conducted to ensure that there was no gross violation of the assumptions of normality, linearity, homogeneity of variances, or homogeneity of regression slopes. The results of ANCOVA are presented as raw means and standard deviations with 95% confidence intervals for the least squares adjusted means and for their differences. The percentage of patients with a decreased ratio at 1 month was compared between the gliadel and non-gliadel groups using Fisher's exact test.

The level of significance was set at 5%. The statistical analysis was conducted with SAS 9.2 for Windows (SAS Institute Inc., Cary, NC, USA).

## 3. Results and Discussion

First of all, data were analysed considering the completeness of the resection. Based on the surgeon's intraoperative opinion and CT early after surgery, 15 patients were judged to have undergone a complete resection, while 8 were judged as partial resections. MIBI SPET showed a mean ratio of 5.9 (min 2.42, max 9.39) for complete resections and a mean ratio of 11.74 (min 6.97, max 16.52) for partial resections. After adjusting for ratio at the baseline, statistical comparison (between complete and partial resections) showed a marginally significant difference between the two groups (*F*_1,20_ = 4.24, *P* = 0.0528) (Table 2).

This marginally significant difference could be due to a number of reasons. First of all, the criteria used (surgeon's opinion and CT very early after surgery) to establish whether or not a resection was complete. More sophisticated methods, such as positron emission tomography/computed tomography (PET/CT), using labelled amino acids (^18^F-FET, ^11^C-methionine) or nucleosides (^18^F-FLT), could have been used, but these tracers (and particularly those with a short half-life) are currently only available at a limited number of units. In addition, HGG is a very aggressive neoplasm that may relapse very soon after surgery, so a significant uptake apparent from the MIBI ratio early after surgery could be genuine neoplasm and not a false-positive finding. Only biopsy could definitively clarify the matter, but this is not done routinely soon after surgery. On the other hand, the mean ratios for partial resections early after surgery were nearly twice as high as for complete resections (11.74 versus 5.9), indicating a greater cellularity in partial resections, as was to be expected.

Then, the effect of gliadel implantation was analysed (Table 3). Although the mean values for the MIBI ratio were significantly higher for patients not treated with gliadel, the effect of the treatment was not statistically significant after adjusting for ratio at the baseline (*F*_1,20_ = 2.63, *P* = 0.1204).

It is worth noting, however, that 100% of the patients treated with gliadel had a significantly decreased ratio early after surgery (mean decrease 59.7%; min 18.4%, max 95.5%), while this was true for only 62.5% of patients in the non-gliadel group (mean decrease 73.3%; min 50.8%; max 90.1%) (*P* = 0.0316), while the other 37.5% of the patients in this latter group had a significant increase (mean increase 81.9%; min 64.7%, max 110.7%) in MIBI uptake 1.5 months (early) after surgery, which was a sign of relapsing disease.

Despite the limited number of patients involved, these data seem to suggest a role for the MIBI ratio in monitoring the early effects (within 2 months) of BCNU, which is known to release its chemotherapeutic agent over a period of 2-3 weeks.

When the MIBI ratio at 4 months was compared with the ratio at one month, all but 2 of the patients (one in the gliadel and one in the non-gliadel group) revealed a significant increase in their MIBI ratio despite receiving chemotherapy, meaning that the disease had already begun to relapse in most patients (86.6%).

When the different behaviour of the MIBI ratio after gliadel application in patients with a first diagnosis vis-à-vis those with recurrent disease (Table 4) was analysed, after adjusting for ratio at the baseline, no statistically significant effect of diagnosis (first versus relapsing disease) came to light (*F*_1,12_ = 0.32, *P* = 0.5797).

## 4. Conclusions

Despite the limited number of patients considered, our data seem to suggest that the MIBI ratio could be a valuable tool for use soon after surgical resections to monitor the effects of chemotherapeutic agents in implanted gliadel wafers. Encouraging preliminary results seem to suggest that MIBI could also be used after gliadel application to rule out any presence of a residual/relapsing neoplastic tissue.

## Figures and Tables

**Table 1:** Patients and data considered in the statistical analysis.

Age	Sex	Completeness of resection	First diagnosis/ recurrent disease	Gliadel (yes/no)	MIBI ratio BS	MIBI ratio early AS	MIBI ratio 4-month AS
69	M	Yes	FD	Yes	24.3	3.5	7.9
64	M	Yes	RD	Yes	23.0	1.0	1.4
58	M	Yes	RD	Yes	19.6	10.0	//
45	M	No	RD	Yes	46.1	15.7	//
58	M	Yes	FD	Yes	14.9	2.4	1.4
59	M	No	FD	Yes	11.0	5.5	//
54	M	Yes	RD	Yes	21.0	3.1	3.4
68	M	Yes	RD	Yes	13.6	10.2	10.3
46	M	No	RD	Yes	14.2	11.6	//
54	M	Yes	RD	Yes	11.1	3.6	//
51	F	Yes	RD	Yes	39.7	9.2	//
58	M	No	FD	Yes	12.5	4.8	5.6
39	F	Yes	RD	Yes	2.4	1.9	//
62	F	No	FD	Yes	17.3	6.9	27.9
66	M	Yes	FD	Yes	17.3	8.4	10.9
51	M	Yes	FD	No	5.6	9.5	19.3
74	M	No	FD	No	14.0	29.4	29.8
75	F	No	FD	No	10.6	17.4	9.0
62	M	Yes	FD	No	40.8	20.1	//
57	M	No	FD	No	11.6	1.2	2.4
72	F	Yes	FD	No	24.0	3.1	6.0
57	M	Yes	FD	No	15.9	2.3	29.7
84	F	Yes	FD	No	3.6	1.7	4.1

FD: first diagnosis; RD: recurrent disease; BS: before surgery; AS: after surgery.

**Table 2:** Comparison of MIBI ratio between completely and partially resected cases.

Complete resection	Raw mean (SD)		Least squares adjusted mean (95% CI)	
	Baseline	EAS	EAS	Yes versus no
Yes (*n* = 15)	18.45 (11.30)	6.00 (5.17)	5.91 (2.42–9.39)	5.84 (−0.078–11.75)
No (*n* = 8)	17.16 (11.91)	11.57 (9.11)	11.74 (6.97–16.52)	

Baseline: MIBI ratio at baseline; EAS: MIBI ratio early after surgery.

**Table 3:** Comparison of MIBI ratio between the gliadel and non-gliadel groups.

Gliadel	Raw mean (SD)		Least squares adjusted mean (95% CI)	
	Baseline	EAS	EAS	Yes versus no
Yes (*n* = 15)	19.21 (11.12)	6.53 (4.20)	6.26 (2.64–9.88)	4.81 (−1.37–10.99)
No (*n* = 8)	15.75 (11.92)	10.57 (10.59)	11.07 (6.10–16.04)	

Baseline: MIBI ratio at baseline; EAS: MIBI ratio early after surgery.

**Table 4:** Comparison of the MIBI ratio between gliadel-treated patients with a first diagnosis as opposed to relapsing disease.

Diagnosis	Raw mean (SD)		Least squares adjusted mean (95% CI)	
	Baseline	EAS	EAS	Yes versus no
First diagnosis (*n* = 6)	16.22 (4.71)	5.27 (2.20)	5.83 (2.40–9.26)	−1.17 (−5.64–3.30)
Relapsing disease (*n* = 9)	21.20 (13.84)	7.37 (5.10)	7.00 (4.21–9.78)	

Baseline: MIBI ratio at baseline; EAS: MIBI ratio early after surgery.
